# Unveiling the Role of Two Rhodopsin-like GPCR Genes in Insecticide-Resistant House Flies, *Musca domestica*

**DOI:** 10.3390/ijms251910618

**Published:** 2024-10-02

**Authors:** Juanjuan Xin, Dylan Brown, Yifan Wang, Xin Wang, Ming Li, Ting Li, Nannan Liu

**Affiliations:** Department of Entomology and Plant Pathology, Auburn University, Auburn, AL 36849, USA; xinjj1104@126.com (J.X.); djb0094@auburn.edu (D.B.); yzw0093@auburn.edu (Y.W.); xzw0083@auburn.edu (X.W.); liming83@mail.sysu.edu.cn (M.L.); tli@alasu.edu (T.L.)

**Keywords:** house flies, insecticide resistance, G-protein coupled receptors (GPCRs), gene mapping, cytochrome P450s

## Abstract

Insecticide resistance in insects, driven by the overexpression of P450 enzymes, presents a significant challenge due to the enhanced metabolic detoxification of insecticides. Although the transcriptional regulation of P450 genes is not yet fully understood, G-protein-coupled receptor (GPCR) genes have emerged as key regulators in this process. This study is the first to associate GPCR genes with insecticide resistance in *Musca domestica*. We identified two key rhodopsin-like GPCR genes, *ALHF_02706.g1581* and *ALHF_04422.g2918*, which were significantly overexpressed in the resistant ALHF strain compared to sensitive strains. Notably, both *ALHF_02706.g1581* and *ALHF_04422.g2918* were mapped to autosome 2, where critical but unidentified regulatory factors controlling resistance and P450 gene regulation are located. This supports our hypothesis that GPCRs function as trans-regulatory factors for P450-mediated resistance. Functional analysis using transgenic *Drosophila* demonstrated that overexpression of these rhodopsin-like GPCR genes increased permethrin resistance by approximately two-fold. Specifically, *ALHF_02706.g1581* overexpression significantly upregulated the *Drosophila* resistance-related P450 genes *CYP12D1*, *CYP6A2*, and *CYP6A8*, while *ALHF_04422.g2918* increased *CYP6G1* and *CYP6A2* expression, thereby enhancing insecticide detoxification in rhodopsin-like GPCR transgenic *Drosophila* lines. These findings suggest that these rhodopsin-like GPCR genes on autosome 2 may act as trans-regulatory factors for P450-mediated resistance, underscoring their critical role in insecticide detoxification and resistance development in *M. domestica*.

## 1. Introduction

The house fly, *Musca domestica*, is a significant domestic, medical, and veterinary pest that serves as a vector for over 200 human and animal pathogens [1,2,3]. Efforts to manage house fly populations have primarily relied on the application of insecticides [4]. However, the development of resistance to these insecticides has posed a major challenge to effective fly management [5,6,7]. Due to its rapid development of resistance and cross-resistance, well-established linkage maps for five autosomes and two sex chromosomes (X and Y), relatively well-understood biochemistry and genetics of insecticide resistance, and the availability of transcriptome and genome sequences [8,9,10], the house fly has been a valuable model for studying resistance mechanisms.

Insect P450s play a critical role in the detoxification of insecticides. Building on our previous transcriptome and gene functional studies in house flies [8,11], we identified several overexpressed P450s as the major functional group involved in house fly resistance. The overexpression of P450s results in elevated levels of P450 proteins and activities, leading to enhanced metabolic detoxification of insecticides. This process is recognized as one of the most important mechanisms of insecticide resistance in insects [7,12,13]. Numerous studies have shown that the overexpression of P450 genes is regulated by trans- or cis-regulatory factors [8,11,14,15,16,17,18,19,20,21,22]. Identifying and characterizing these regulatory factors is crucial for advancing our understanding of the development of insecticide resistance in insects.

G-protein-coupled receptors (GPCRs) interact with G proteins, which subsequently activate downstream regulatory factors such as the G-protein alpha subunit (Gαs), adenylate cyclase (AC), cyclic adenosine monophosphate (cAMP), and protein kinase A (PKA), thereby regulating various physiological processes [23,24,25]. In insects, GPCRs have been reported to regulate growth and development [26], and they have been implicated in stress responses to desiccation [27] and blood-feeding behavior [28]. However, the downstream factors in these GPCR pathways are not yet fully understood. The role of GPCRs in insecticide resistance remains largely unexplored, and no insecticides have been specifically developed to target GPCRs. [29,30,31,32] reported the overexpression of a GPCR gene in a highly pyrethroid-resistant mosquito *Culex quiquenfaciatus*, and demonstrated the involvement of this GPCR and its downstream regulatory factors (Gαs, AC, cAMP, and PKA) in regulating the expression of resistance-related P450 genes, ultimately leading to P450-mediated insecticide resistance in resistant mosquitoes [29,30,33,34,35]. Employing whole transcriptome analyses [8,11] identified multiple P450 genes and GPCR genes that were overexpressed in pyrethroid-resistant house flies. This finding led to the proposal that these overexpressed GPCR genes might play a role in regulating P450-mediated resistance in house flies.

Building on our earlier transcriptome data analysis, which involved multiple detoxification genes—primarily P450s—and signaling regulatory genes of GPCRs in resistant house flies [8], our characterization of the role of P450 overexpression in resistance [11], and our discovery of cis/trans factors in the co-regulation of resistance-related P450 gene expression in resistant house flies [11], the current study aimed to explore the potential regulatory interaction between GPCR overexpression and P450 gene expression, which plays a crucial role in mediating insecticide resistance in house flies. We assessed the expression of two rhodopsin-like GPCR genes in both resistant and susceptible house flies and conducted in vivo functional studies of these GPCRs in P450-mediated resistance using transgenic *Drosophila melanogaster* lines. We further examined the relative expression of four resistance-related P450 genes in the GPCR transgenic *Drosophila* lines to pinpoint the role of these rhodopsin-like GPCRs in the regulation of resistance-related P450s. Furthermore, we conducted autosomal mapping of the overexpressed rhodopsin-like GPCR genes on Muller elements [36] to investigate the linkage of these rhodopsin-like GPCRs to trans-regulatory factors on autosome 2, which has been shown to play a role in the regulation of P450 gene expression in resistant house flies. We hypothesized that GPCRs function as trans-regulatory factors for P450-mediated resistance.

## 2. Results

### 2.1. Rhodopsin-like GPCR Gene Up-Regulation in Permethrin Resistant House Fly, M. domestica

In the current study, we built upon previous transcriptome analysis findings [8] that identified multiple genes, including metabolic detoxification P450 genes and signaling transduction GPCR genes, as overexpressed in resistant house flies. Additionally, linkage analysis revealed that these overexpressed resistance-related P450s are regulated by trans-regulatory factors on autosome 2 [11]. To further explore these findings, we examined the expression levels of the identified GPCR genes, *ALHF_02706.g1581* and *ALHF_04422.g2918*, in house fly strains using quantitative real-time polymerase chain reaction (qRT-PCR) with specific primer pairs for each gene (Table 1).

**Figure 1:**
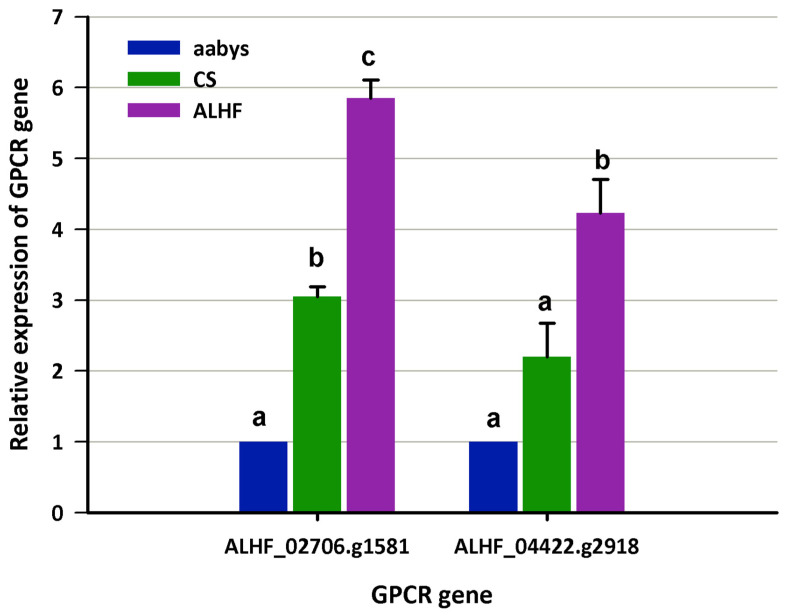
The expression of *GPCR* genes in house flies, *Musca domestica*. The expression levels of GPCR genes *ALHF_02706.g1581* and *ALHF_04422.g2918* were assessed in the susceptible house fly strains aabys and CS, as well as in a resistant strain. mRNA levels of both GPCR genes were quantitatively measured using qRT-PCR. Results are presented as mean ± S.E. (*n* ≥ 3). Statistical significance in gene expression among samples, indicated by different letters (a, b, or c), was determined using one-way ANOVA with a *p*-value ≤ 0.05.

We compared the expression levels of these GPCR genes between the resistant ALHF strain and the susceptible aabys and CS strains. This approach was chosen to avoid false positive results that could arise from analyzing mosquitoes of different genetic backgrounds or inherent tolerances, rather than true resistance. qRT-PCR results confirmed that two GPCRs, *ALHF_02706.g1581* and *ALHF_04422.g2918*, were significantly overexpressed in the ALHF-resistant strain compared to the two susceptible strains (Figure 1). Specifically, the relative expression levels of *ALHF_02706.g1581* and *ALHF_04422.g2918* were upregulated 5.9-fold and 4.2-fold, respectively, in ALHF compared to aabys, and 1.9-fold compared to the CS strain (Figure 1). These two GPCRs were the focus of further characterization to understand their roles in insecticide resistance in house flies.

### 2.2. Localization and Mapping of Overexpressed GPCR Genes to the Autosomes of House Flies

The landmarks of Muller elements [37], known as the six highly conserved chromosomal arms of the well-annotated *Drosophila* genome [38], have enabled researchers to map genes from other species to autosomes [39]. Muller elements A–E are autosomal, while F is linked to the X chromosome. Meisel and Scott [36] have accurately mapped *M. domestica* genes to Muller elements, with Muller element A corresponding to house fly chromosome 3, B to chromosome 1, C to chromosome 5, D to chromosome 4, E to chromosome 2, and F to the X chromosome [36]. Using a similar methodology, we performed BLAST analysis [40] of two overexpressed GPCR genes, ALHF_02706.g1581 and ALHF_04422.g2918, against *D. melanogaster* sequences. The results showed that ALHF_02706.g1581 and ALHF_04422.g2918 aligned with the *Drosophila* homologs *Dm Rh1* and *Dm Rh2*, respectively. Therefore, we refer to these two GPCR genes as rhodopsin-like GPCRs. These rhodopsin-like GPCR genes are located on chromosome 3R (Muller element E), which corresponds to autosome 2 of house flies [36] (Table 2).

**Figure 2:**
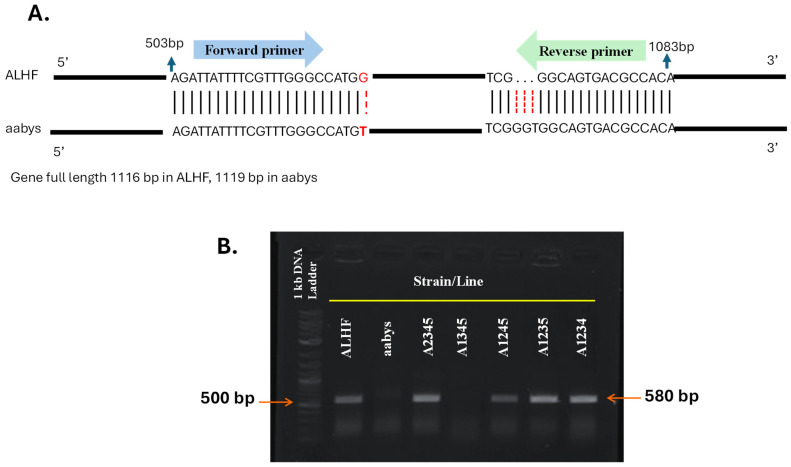
Allele-specific RT-PCR for autosomal mapping of GPCR gene *ALHF_02706.g1581* in *M. domestica*. The full length of the GPCR gene *ALHF_02706.g1581* is 1116 bp in both the ALHF and aabys strains. (**A**), PCR fragments were generated using an allele-specific primer set designed according to the ALHF sequence of *ALHF_02706.g1581*. (**B**). The absence of a PCR product in a house fly line indicates that the gene is located on the corresponding autosome from aabys (e.g., the absence of a band in the A1345 line indicates that the gene is located on autosome 2).

Validation of the autosomal location of the rhodopsin-like GPCR gene was further conducted using allele-specific PCR [19]. The rhodopsin-like GPCR gene *ALHF_02706.g1581* was chosen for validation due to the presence of single nucleotide polymorphisms (SNPs) between the resistant ALHF strain and the aabys strain. In contrast, the *ALHF_04422.g2918* gene showed identical sequences in both strains. The allele-specific PCR primers for the rhodopsin-like GPCR gene *ALHF_02706.g1581* were designed based on the ALHF sequence, incorporating a specific SNP at the 3′ end of one primer to enable preferential amplification of ALHF alleles (Table 2, Figure 2A) [11,41]. Our results indicate that the ALHF allele-specific primer sets for *ALHF_02706.g1581* successfully amplified specific DNA fragments (~580 bp) exclusively in flies carrying the wild-type autosome 2 markers from ALHF. No amplification occurred in flies homozygous for autosome 2 from either the aabys strain or the A1345 strain (Figure 2B). 

PCR product-sequence analysis indicated that the amplified fragment corresponds to the house fly gene *ALHF_02706.g1581*. These findings confirm the mapping of the rhodopsin-like GPCR gene to autosome 2.

### 2.3. Up-Regulated House Fly Rhodopsin-like GPCR Genes in Transgenic D. melanogaster—An Approach for Defining the House Fly Rhodopsin-like GPCR Gene Function

**Figure 3:**
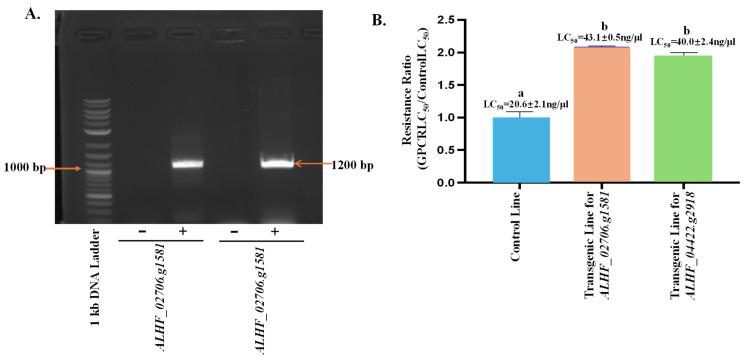
Effects of overexpressed GPCR genes on permethrin resistance and P450 gene expression in *Drosophila melanogaster*. (**A**) RT-PCR amplification of the GPCR genes *ALHF_02706.g1581* and *ALHF_04422.g2918* in transgenic *Drosophila melanogaster* lines. “−” indicates the non-transgenic control *D. melanogaster* line, while “+” represents the transgenic *D. melanogaster* lines containing house fly GPCR genes. The 1 kb DNA Plus ladder (Biolabs) was used as the molecular size reference, with numbers indicating DNA band sizes in bp. (**B**) Toxicity of permethrin to non-transgenic (control) and transgenic *Drosophila melanogaster* lines. Resistance ratios were calculated as LD50 of *D. melanogaster* lines/LD50 of the control line. Statistical significance in gene expression among samples, indicated by different letters (a, b, or c), was determined using one-way ANOVA with a *p*-value ≤ 0.05. No significant differences in expression levels were observed between groups labeled with the same letter (*p* < 0.05).

To characterize the function of the up-regulated genes *ALHF_02706.g1581* and *ALHF_04422.g2918* in the development of insecticide resistance in ALHF house flies, we conducted a transgenic study using the GAL4-UAS enhancer trap system in *D. melanogaster*. To confirm the presence of house fly rhodopsin-like GPCR genes in the transgenic *Drosophila* lines, we performed RT-PCR using full-length primer pairs for *ALHF_02706.g1581* and *ALHF_04422.g2918* (Table 1), and cDNA from three *Drosophila* lines: the control (F1 progeny of Bloomington stock #24484 *D. melanogaster* line containing an empty pUAST vector crossed with GAL4), transgenic *ALHF_02706.g1581* (F1 progeny of homozygous transgenic *ALHF_02706.g1581* crossed with GAL4), and transgenic *ALHF_04422.g2918* (F1 progeny of homozygous transgenic *ALHF_04422.g2918* crossed with GAL4). The results showed that the full lengths of *ALHF_02706.g1581* and *ALHF_04422.g2918* were only amplified in the *ALHF_02706.g1581* and *ALHF_04422.g2918* transgenic lines, respectively, with specific gene sizes ~1200 bp (Figure 3A), corresponding to the size of ~1200 bp for these two rhodopsin-like GPCR genes. Sequencing of the PCR products from these transgenic lines confirmed that they are indeed the full lengths of rhodopsin-like GPCR *ALHF_02706.g1581* and *ALHF_04422.g2918*.

The sensitivity of the *D. melanogaster* transgenic lines to permethrin was assessed through bioassays conducted on three *Drosophila* lines: the control (CK), *ALHF_02706.g1581*, and *ALHF_04422.g2918*. The transgenic lines *ALHF_02706.g1581* and *ALHF_04422.g2918* exhibited 2.1-fold and 2.0-fold increases, respectively in permethrin tolerance, compared to the control line, as measured by the LC50 values (Figure 3B, *p* = 0.005). These results suggest that the up-regulated rhodopsin-like GPCR genes in the house fly enhance permethrin resistance in D. melanogaster, underscoring the significant role of these genes in permethrin resistance in M. domestica.

### 2.4. Relative Expression Levels of Drosophila P450 Genes in House Fly Rhodopsin-like GPCR Transgenic Lines 

**Figure 4:**
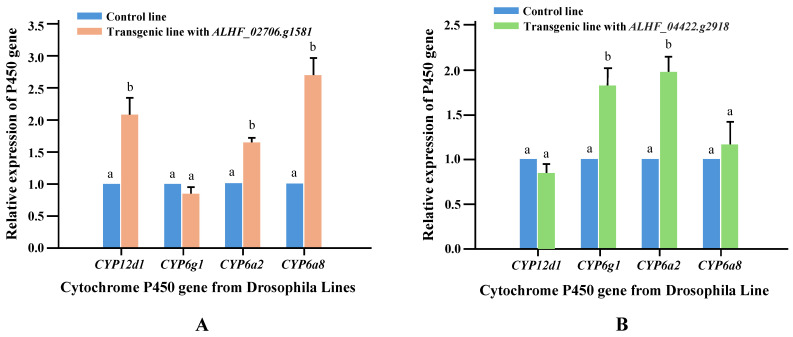
Relative expression levels of four *Drosophila* resistance-related P450 genes in control and GPCR transgenic lines. The expression patterns of four P450 genes (CYP6A2, CYP6A8, CYP12D1, CYP6G1) were analyzed in control and transgenic *Drosophila* lines using qRT-PCR. (**A**) Expressing of the GPCR genes *ALHF_02706.g1581* and (**B**) Expressing of the GPCR *ALHF_04422.g2918*. The relative expression levels in transgenic flies are shown relative to their expression in the control line. Results are presented as mean ± S.E. (*n* ≥ 3). Statistical significance in gene expression among samples, indicated by different letters (a or b), was determined using one-way ANOVA with *p* ≤ 0.05.

Li et al. [29] identified that resistance-related GPCRs can induce the overexpression of *Drosophila* P450s, which are known to be involved in insecticide resistance, and proposed that the molecular mechanism by which GPCRs contribute to insecticide resistance in transgenic *Drosophila* involves the regulation of these resistance P450 genes. To fully understand the role of GPCRs in permethrin resistance in transgenic *Drosophila*, we conducted qRT-PCR to measure the relative expression levels of four *Drosophila* resistance P450 genes (*CYP12D1*, *CYP6G1*, *CYP6A2*, and *CYP6A8*), which are associated with insecticide resistance in *Drosophila* [42]. The qRT-PCR was performed using cDNA from the control (CK), *ALHF_02706.g1581*, and *ALHF_04422.g2918* transgenic *Drosophila* lines with primer pairs designed according to the sequences of *CYP6A2*, *CYP6A8*, *CYP12D1*, and *CYP6G1* (Table 1). Significant increases (*p* < 0.001) in the expression of *CYP12D1*, *CYP6A2*, and *CYP6A8* (2.1-, 1.7-, and 2.7-fold, respectively) were observed in the *ALHF_02706.g1581* transgenic line compared to the control line (Figure 4A). In the *ALHF_04422.g2918* transgenic line, increased expression levels were found for *CYP6G1* (1.8-fold) and *CYP6A2* (2.0-fold) (Figure 4B).

## 3. Discussion

The overexpression of P450 enzymes leads to elevated protein levels and enhanced metabolic detoxification of insecticides, establishing P450 detoxification as a key mechanism in insecticide resistance among insects. While our understanding of the regulation of insect P450 gene expression remains incomplete, studies have revealed the complex nature of these transcriptional regulation mechanisms [14,20,21,22,34,35,43,44]. Known regulatory factors include orphan nuclear receptors, Cap ‘n’ Collar C (CncC)/muscle aponeurosis fibromatosis (Maf), GPCRs, and cAMP-response element binding proteins, identified across various insect species [14,29,35,44]. For instance, Hu et al. [45] reported that the upregulation of CncC/Maf transcription factors contributes to the increased expression of *CYP321A8* in a resistant strain of *Spodoptera exigua*, facilitated by specific promoter site mutations. Similarly, Yang et al. [44] demonstrated that the overexpression of *CYP6CM1* in whiteflies, which confers resistance to neonicotinoid insecticides, is regulated by CREM, activated via the MAPK pathway. These findings underscore the intricate mechanisms involved in the transcriptional regulation of insect P450 genes, which are crucial for detoxifying insecticides and developing resistance.

GPCR genes and signaling pathways play a pivotal role in regulating P450 genes associated with resistance in several insect species, including Lymantria dispar [46], Culex pipiens [47], Culex quinquefasciatus [29,30], and M. domestica [48]. GPCR pathways have emerged as significant regulators of P450 gene expression. The GPCR regulatory pathway involving Gαs, adenylate cyclase (AC), protein kinase A (PKA), and cyclic AMP (cAMP) in mosquitoes has been systematically studied and modeled [29,30,34,35], showing its involvement in regulating multiple resistance-related P450 gene expressions. This pathway contributes to enhanced insecticide detoxification in Culex quinquefasciatus. Functional studies by Li et al. [29,30,35], using transgenic Drosophila, demonstrated that rhodopsin-like GPCRs increase permethrin tolerance and elevate the expression of resistance P450 genes CYP12D1 and CYP6A8 of Drosophila melanogaster. Additional studies using transgenic Drosophila lines further confirm the involvement of GPCRs in regulating P450 gene expression and insecticide resistance in L. dispar and M. domestica.

Our results demonstrate that the two rhodopsin-like GPCR genes, ALHF_02706.g1581 and ALHF_04422.g2918, differentially regulate cytochrome P450 (CYP) genes, providing important insights into the molecular mechanisms underlying insecticide resistance. Specifically, *ALHF_02706.g1581* primarily upregulated the resistance-related P450 genes *CYP12D1*, *CYP6A2*, and *CYP6A8*, while ALHF_04422.g2918 upregulated *CYP6G1* and *CYP6A2*. The differential regulation of these CYP genes by distinct GPCRs indicates not only that resistance mechanisms in house flies are highly specialized, with specific GPCRs selectively enhancing P450 gene expression depending on the insecticide, but also that these GPCRs may be involved in distinct signaling pathways. This specificity underscores the complexity of GPCR-mediated regulatory networks and highlights the potential for targeting these pathways in future strategies to combat insecticide resistance. CYP6A2, CYP6A8, CYP6G1, and CYP12D1 are known to be involved in the detoxification of various insecticides, including organophosphates, pyrethroids, DDT, and neonicotinoids [42,49,50]. The upregulation of these P450 genes by ALHF_02706.g1581 and ALHF_04422.g2918 could suggest a role for the GPCR genes in enhancing resistance to these compounds by facilitating the breakdown of insecticides. Notably, the upregulation of *CYP6G1* and *CYP6A2* by ALHF_04422.g2918 is particularly significant, as *CYP6G1* has been widely implicated in resistance to multiple insecticides, including DDT and neonicotinoids, due to its broad substrate specificity. The high expression of *CYP6G1* in insecticide-resistant *Drosophila* and other insect species suggests that ALHF_04422.g2918 plays a key role in promoting resistance to a diverse range of insecticides.

Both trans- and cis-regulatory factors have been implicated in the overexpression of P450 genes in resistant insects. The overexpression of *CYP6A1* and *CYP6D1* in the house fly *M. domestica*, and *CYP6A2* and *CYP6A8* in the fruit fly *D. melanogaster*, is trans-regulated by factors on autosome 2 [15,18,19], although the precise regulatory factors remain undefined. Similar trans-regulation of P450-mediated resistance has been proposed in the mosquito *Aedes aegypti* [22] but the regulatory factors have yet to be identified. A recent systematic study at the whole transcriptome level on insecticide-resistant house flies aimed to develop a deeper understanding of how P450 gene upregulation, interaction, metabolism, and function contribute to the development of insecticide resistance [8,11]. Combining in vivo functional studies using the *D. melanogaster* transgenic system, in vitro functional metabolism studies, in silico homology modeling, and molecular docking methods, Li Ming et al. [11] demonstrated that the overexpression of P450 genes in house flies contributes to pyrethroid resistance through increased insecticide metabolism and multiple P450 interactions and regulation. Genetic linkage analysis further revealed that the overexpression of these P450 genes is regulated by trans- and/or cis-acting factors, particularly on autosome 2, consistent with findings in other insect strains or species.

Our current research is progressing towards highlighting the significant role of G-protein-coupled receptor (GPCR) gene overexpression in regulating P450-mediated insecticide resistance in house flies. Our findings of increased permethrin resistance in transgenic *Drosophila* expressing house fly rhodopsin-like GPCR genes, consistent with findings in *Culex* mosquitoes, further confirm the role of GPCRs in insecticide resistance. The increased expression of specific resistance-related *Drosophila* P450 genes (*CYP12D1*, *CYP6A2*, *CYP6A8*, *CYP6G1*) [42] induced by overexpressed rhodopsin-like GPCR genes in transgenic *Drosophila* underscores the role of rhodopsin-like GPCRs in regulating these P450 genes, thereby enhancing insecticide detoxification. Furthermore, genome mapping of these overexpressed rhodopsin-like GPCR genes on autosome 2 in house flies, confirmed through Muller element analysis and allele-specific PCR, has pinpointed their location corresponding to that of regulatory factors [11]. These findings support our hypothesis that overexpressed GPCRs act as trans-regulatory factors for P450-mediated insecticide resistance in house flies. Nevertheless, the precise regulatory role of GPCRs in house fly P450 gene expression, and the full spectrum of factors involved in GPCR pathways responsible, together with GPCR, for the development of resistance, remain to be fully elucidated. 

## 4. Materials and Methods

### 4.1. House Flies 

Three house fly strains—ALHF, aabys, and CS—were used in this study. ALHF was originally collected from a poultry farm in Alabama in 1998. This strain exhibited a high level of resistance after being selected with permethrin for six generations and has been annually selected with permethrin to maintain its resistance level (~1100-fold) [5,51]. The aabys strain is insecticide-susceptible and carries five recessive morphological markers: ali-curve (ac), aristapedia (ar), brown body (bwb), yellow eyes (ye), and snipped wings (snp), distributed on autosomes 1, 2, 3, 4, and 5, respectively. CS is a wild-type insecticide-susceptible strain that has been kept in laboratory breeding for over five decades, sharing the same morphological characteristics as ALHF. The aabys and CS strains were originally obtained from Dr. J. G. Scott (Cornell University).

Five backcross (BC1) lines were genetically isolated as described by Liu and Yue [5] and Tian et al. [51]. Briefly, approximately 400 ALHF virgin females were crossed with aabys males to generate the first filial generation (F1). Since crossing over rarely occurs in male flies [52], the F1 males (about 200 flies) were backcrossed with same similar amount of aabys virgin females. This ensured that the presence of a mutant phenotype indicated that the respective autosome with a mutant-type marker was derived from aabys. Five homozygous back-cross (BC1) lines were isolated with the following genotypes: ac/ac, +/a+, +/+, +/+, +/+ (A2345); +/a+, ar/ar, +/+, +/+, +/+ (A1345); +/a+, +/a+, bwb/bwb, +/+, +/+ (A1245); +/+, +/+, +/+, ye/ye, +/+ (A1235); and +/+, +/+, +/+, +/+, snp/snp (A1234). The name of each line indicates which of its autosomes has a wild-type marker from ALHF. For example, the A2345 strain has wild-type markers on autosomes 2, 3, 4, and 5 from ALHF and the mutant marker (ali-curve) on autosome 1 from aabys. All house flies were reared at 25 ± 2 °C under a photoperiod of 12:12 (L:D) h.

### 4.2. RNA Extraction and cDNA Preparation

Twenty three-day-old virgin female house flies from each of the three strains (ALHF, aabys, and CS) and five BC1 lines were collected for RNA extraction. Total RNA was isolated using the acidic guanidine thiocyanate-phenol-chloroform method [53]. Following extraction, DNA was removed from 5 μg of total RNA using the TURBO DNA-free kit (Ambion), according to the manufacturer’s instructions. cDNA synthesis was performed using the ProtoScript^®^ II First Strand cDNA Synthesis Kit (New England Biolabs, Ipswich, MA, USA). The resulting cDNA was quantified using a Nanodrop spectrophotometer before proceeding to qRT-PCR and PCR analyses. Each experiment was independently replicated more than three times, involving separate insect sample collections, RNA extractions, and cDNA syntheses.

### 4.3. Quantitative Real-Time Polymerase Chain Reaction (qRT-PCR)

qRT-PCR was performed using the SYBR Green PCR Master Mix kit and an ABI 7500 Quantitative PCR System (Applied Biosystems, Foster City, CA, USA). Each qRT-PCR reaction (25 µL) contained 1x SYBR Green master mix, 1 µL of cDNA, and a GPCR gene-specific primer pair (Table 1) at a final concentration of 3–5 µM. The β-actin gene, an endogenous control [54], was used as it remains constant across different tissues and in both resistant and susceptible house flies [8,11,41,55]. A “no-template” reaction served as a negative control. All samples were run in triplicate. The reaction cycle included an initial melting step at 50 °C for 2 min, followed by 95 °C for 10 min, and then 40 cycles of 95 °C for 15 s and 60 °C for 1 min. The PCR reaction was assessed by a melting curve analysis using Dissociation Curves software. Relative expression levels for specific genes were calculated using the 2^−ΔΔCT^ method with SDS RQ software v.2.3 [56]. Each experiment was repeated three times with different cDNA samples.

The statistical significance of gene expression was calculated using a Student’s *t*-test for all two-sample comparisons and a one-way analysis of variance (ANOVA) for multiple sample comparisons, utilizing the Statistical Package for the Social Sciences (SPSS) software with both least significant difference (LSD) and Tukey tests to analyze the significance of means. A *p*-value ≤ 0.05 was considered statistically significant. Significant up-regulation was determined using a cut-off value of a two-fold change in expression [57].

### 4.4. Autosome Assignment and Mapping of Overexpressed GPCR Genes in M. domestica

*Drosophila* genomes are organized into six chromosomal arms, known as Muller elements [37], where Elements A–E are autosomal, and Element F corresponds to the ancestral X chromosome of Brachycera [58,59]. Mapping genes to these chromosome arms in *Drosophila* [39] facilitates the assignment of homologous genes from other species to these Muller elements. We mapped our GPCR gene sequences, *ALHF_02706.g1581* (NCBI accession number: XP_005182983.1) and *ALHF_04422.g2918* (NCBI accession number: XP_005191160.1), to homologous genes in the *Drosophila* genome, thereby assigning them to specific Muller elements. We then aligned these Muller elements to the corresponding house fly autosomes according to [36].

Allele-specific PCR was conducted to further validate the linkage mapping of the GPCR in house flies. GPCR gene *ALHF_02706.g1581* was selected because its sequence showed nucleotide polymorphisms (SNPs) within the gene between the resistant ALHF and the aabys strains, whereas GPCR gene *ALHF_04422.g2918* displayed identical sequences between the two strains. In this experiment, two house fly strains (ALHF and aabys) and five BC1 lines (A2345, A1345, A1245, A1235, and A1234) were used. Each BC1 line was homozygous for the recessive mutant allele from aabys and heterozygous for the dominant wild-type alleles from ALHF. Allele-specific PCR was performed using a two-round PCR strategy as described by Liu and Scott [19]. Briefly, the first PCR reaction was conducted using allele-independent primer pairs (Table 1) to generate GPCR cDNA fragments. This was followed by a second PCR reaction containing 0.5 µL of the first-round PCR reaction solution and the allele-specific primer pair (Table 1), which was designed based on the specific sequence of the genes from ALHF, with a specific nucleotide polymorphism at the 3’ end of the primer to allow preferential amplification of the allele from ALHF. Three biological replicates were conducted with different mRNA samples, and the PCR products were sequenced.

### 4.5. Amplification of Full Length of GPCR Genes

To study the function of GPCR genes, the full-length sequences of genes *ALHF_02706.g1581* and *ALHF_04422.g2918* were generated using Platinum Taq DNA Polymerase High-Fidelity (Invitrogen, Carlsbad, CA, USA) with cDNA from ALHF as the template. The PCR primers for full-length gene amplification were designed based on sequences from previous transcriptome data of the ALHF house fly strain. The forward primers included protecting bases CCG and an EcoRI restriction enzyme site (GAATTC), while the reverse primers contained CTA and an XbaI sequence (TCTAGA) (Table 1). The PCR process consisted of an initial denaturation at 94 °C for 3 min, followed by 35 cycles of 94 °C for 30 s, 57 °C for 45 s, and 72 °C for 2 min and 30 s, with a final extension at 72 °C for 5 min. PCR products were purified using the QIAquick Gel Extraction Kit (Qiagen, Valencia, CA, USA) before being cloned for sequencing using the TOPO TA Cloning Kit (Invitrogen, Carlsbad, CA) and the One Shot TOPO 10 Chemically Competent E. coli Kit (Invitrogen, Carlsbad, CA). Plasmids from the TA clones were extracted using the E.Z.N.A. Plasmid DNA Mini Kit 1 (OMEGA Bio-tek, Inc., orcross, GA, USA). Cloning and sequence analyses were repeated at least three times and three TA clones from each replication to ensure the accuracy of GPCR cDNAs.

### 4.6. Construction of Transgenic Drosophila Flies

The plasmid containing the full length of each GPCR from the TA clone was digested using restriction enzymes EcoRI/XbaI and sub-cloned into a pUASTattB vector [60,61] [a gift from Dr. Johnnes Bischof, University of Zurich]. Plasmid DNA of pUASTattB-2706 and pUASTattB-4422 was extracted using the Endo-Free Plasmid Purification Kit (Qiagen, Valencia, CA) and were transformed into the germline of *D. melanogaster* (Bloomington stock #24484, genotype M{vas-int.Dm}ZH-2A, M{3xP3-RFP.attP’}ZH-58A), resulting in site-specific integration on chromosome 2R (Rainbow Transgenic Flies Inc., Camarillo, CA, USA) [62]. The following cross-breeding process was conducted as described by Li [29]. After microinjecting the plasmids pUASTattB-2706 and pUASTattB-4422 into fruit fly embryos, transgenic flies exhibiting orange eyes and straight wings were crossed with a W1118 strain (white eyes) to produce heterozygous transgenic *Drosophila* carrying the target GPCR transgenes, characterized by orange eyes and straight wings. These heterozygous transgenic flies were then crossed with a balancer strain with white eyes and curly wings (Bloomington stock #6312; genotype: w [1118]/Dp(1;Y)y[+]; sna[Sco]/CyO, P{ry[+t7.2]=sevRas1.V12}FK1). Offspring with orange eyes and curly wings were self-crossed to generate homozygous transgenic flies. The transgenic lines used for all subsequent assays were the F1 progeny from crosses between homozygous GPCR transgenic virgin females and GAL4-expressing males (ubiquitous Act5C driver line). This resulted in the lines ALHF_02706.g1581+GAL4 and ALHF_04422.g2918+GAL4. “The control group consisted of the F1 progeny from a cross between the control line (Bloomington stock #24484, *D. melanogaster* containing an empty pUAST vector) and GAL4-expressing males.

### 4.7. Toxicity of Permethrin on the Transgenic D. melanogaster

Permethrin toxicity bioassays were performed on 2–3 day post-eclosion female homozygous transgenic *Drosophila* of *ALHF_02706.g1581* and *ALHF_04422.g2918*, and CK to assess their susceptibility to permethrin. Serial concentrations of permethrin solution in acetone, ranging from 12 to 70 ng/μL, were prepared, ensuring mortality rates between 0% and 100% for the tested *Drosophila*. Each concentration (200 μL) was evenly applied to the inner surface of individual 20 mL glass scintillation vials. Fifteen female flies were then placed into each prepared vial. The vials were sealed with cotton balls soaked in 5% sucrose. Control group vials were coated with acetone alone and plugged with identical 5% sucrose-soaked cotton balls. Mortality was assessed after 24 h of exposure to permethrin. Susceptibility to permethrin in transgenic flies was determined by comparing the LC50 values of GPCR transgenic flies to the control group, analyzed using standard probit analysis with a computerized version of [63]. Each bioassay was independently replicated three times. All *Drosophila* were reared on Jazz-Mix *Drosophila* food (Fisher, KS City, MO, USA) at 25 ± 2 °C under a 12:12 (L:D) photoperiod, following standard protocols [64].

### 4.8. Expression of Drosophila Insecticide-Related P450 Genes in the House Fly GPCR Transgenic Drosophila Lines

qRT-PCR was used to detect the relative expression levels of four *Drosophila* resistance P450 genes, *CYP12D1*, *CYP6G1*, *CYP6A2*, and *CYP6A8* [42] in CK, *ALHF_02706.g1581* and *ALHF_04422.g2918* transgenic *Drosophila* lines. Female *Drosophila*, 2–3 days post-eclosion, were collected for the P450 gene expression analysis, following the same qRT-PCR method described previously. The primers used for the PCR reactions for *CYP12D1*, *CYP6G1*, *CYP6A2*, and *CYP6A8* are listed in Table 1.

## 5. Conclusions

The two GPCR genes *ALHF_02706.g1581* and *ALHF_04422.g2918* are significantly overexpressed in the insecticide-resistant strain of *M. domestica* (ALHF). When overexpressed in transgenic *Drosophila* through the GAL4/UAS system, these genes improved the resistance level of *Drosophila* to permethrin and promoted the expression of specific P450 genes. Both *ALHF_02706.g1581* and *ALHF_04422.g2918* mapped on autosome 2, which is associated with the regulation of P450 genes in house flies, indicating that GPCR genes play a critical role in the regulatory pathway of P450 genes in *M. domestica*. This study sheds new light on the potential function of GPCR genes in insecticide resistance in *M. domestica*. Future research should aim to provide more evidence on the function of GPCRs in insecticide resistance development and clarify the exact regulatory pathways involved. Gene knockdown and in vitro gene expression studies are suggested for future functional studies of GPCR genes in *M. domestica*. Understanding the downstream factors in the GPCR regulatory pathway will be crucial for identifying new insecticide targets, supporting the development of novel insecticides, and helping control house fly populations and house fly-borne diseases.

## Figures and Tables

**Table 1:** The list of primers used in the present study.

	Gene Name	GeneBank ID	qRT Primers (5′-3′); F (Forward), R (Reverse)
qRT-PCR	*ALHF_02706.g1581*	LOC101898316	qRT 1581F: CAAAATCTCTGCGTACACCTGC qRT 1581R: AACCGGCATAGACATCACACAT
	*ALHF_04422.g2918*	LOC101888328	qRT 2918F: GTCCTCAATTTGGCCTTTTCCG qRT 2918R: CGATCCAGGGCTATCATACACA
	*CYP6A2*		qRT 6a2F: TGGACGGAAAGAAGTGGAAGGAC qRT 6a2R: AGTTCATGTTCCCGACGGTGATCA
	*CYP12D1*		qRT 12d1F: GCTCGGCTCAAATGTGCTGATGAA qRT 12d1R: TGACCTGCATCTTCTTTCCGGTCT
	*CYP6A8*		qRT 6a8F: ACGAGTGCACCAAGGATCTGAAG qRT 6a8R: ATTGACCAGCCTCGATGACGAAGT
	*CYP6G1*		qRT 6g1F: CGGCTGAAGGACGAGGCTG qRT 6g1R: GCTATGCTGTCCGTGGAGAACTGA
	*β-actin*		actinF: ATGAGGCTCAGAGCAAACGTGG actinR: AGTCATCTTCTCGCGATTGGCCT
Full Length Clone	*ALHF_02706.g1581*	LOC101898316	Full-1581F: CCGGAATTCCAAAATGGAAAGATTCGCTGAACA Full-1581R: CTAGTCTAGATGCCTTTGATTCGGACTCAGT
	*ALHF_04422.g2918*	LOC101888328	Full-2918F: CCGGAATTCCAAAATGAGAGATGACATGGCG Full-2918R: CTAGTCTAGAAGCCTGGGACTCTGCCTGT
Allele-Specific PCR	*ALHF_02706.g1581*	LOC101898316	Allele-F: AGATTATTTTCGTTTGGGCCATGG Allele-R: TGTGGCGTCACTGCCCGA

**Table 2:** Autosome assignments of overexpressed GPCR genes in house flies.

GPCR Gene [8]	Gene_ID	Accession Number	*Drosophila* Chromosome (Mullers Element)	*Drosophila* Homologous Gene	House Fly Autosome [36]
ALHF_02706.g1581	LOC101898316	XP_005182983.1	3R (E)	Dm Rh1 *	2
ALHF_04422.g2918	LOC101888328	XP_005191160.1	3R (E)	Dm Rh2 **	2

* ninaE neither inactivation nor afterpotential E [*Drosophila melanogaster* (fruit fly)]-Gene-NCBI (nih.gov). ** Rh2 Rhodopsin 2 [*Drosophila melanogaster* (fruit fly)]-Gene-NCBI (nih.gov).

## Data Availability

The data that support the findings of this study are available from the corresponding author, N.L., upon request.
